# Rapid diagnosis of *Capnocytophaga canimorsus* septic shock in an immunocompetent individual using real-time Nanopore sequencing: a case report

**DOI:** 10.1186/s12879-019-4173-2

**Published:** 2019-07-24

**Authors:** Seweryn Bialasiewicz, Tania P. S. Duarte, Son H. Nguyen, Vichitra Sukumaran, Alexandra Stewart, Sally Appleton, Miranda E. Pitt, Arnold Bainomugisa, Amy V. Jennison, Rikki Graham, Lachlan J. M. Coin, Krispin Hajkowicz

**Affiliations:** 1 Centre for Children’s Health Research, Children’s Health Queensland, 62 Graham St., South Brisbane, QLD 4101 Australia; 2 Child Health Research Centre, The University of Queensland, 62 Graham St., South Brisbane, QLD 4101 Australia; 3 Institute for Molecular Bioscience, The University of Queensland, 306 Carmody Rd, St Lucia, QLD 4072 Australia; 4 Infectious Diseases Unit Royal Brisbane and Women’s Hospital, Level 6, Joyce Tweddell Building, Royal Brisbane and Women’s Hospital, Brisbane, QLD 4029 Australia; 5 QML Pathology, PO Box 2280, Mansfield, QLD 4122 Australia; 6 School of Clinical Medicine, University of Queensland Level 6, Oral Health Centre, (883) 288 Herston Road, Herston, QLD 4006 Australia; 7 Forensic and Scientific Services, Queensland Department of Health, 39 Kessels Rd, Coopers Plains, QLD 4108 Australia

**Keywords:** Nanopore sequencing, Droplet digital PCR, Capnocytophaga canimorsus, Diagnosis, Sepsis

## Abstract

**Background:**

Rapid diagnosis and appropriate treatment is imperative in bacterial sepsis due increasing risk of mortality with every hour without appropriate antibiotic therapy. Atypical infections with fastidious organisms may take more than 4 days to diagnose leading to calls for improved methods for rapidly diagnosing sepsis. *Capnocytophaga canimorsus* is a slow-growing, fastidious gram-negative bacillus which is a common commensal within the mouths of dogs, but rarely cause infections in humans. *C. canimorsus* sepsis risk factors include immunosuppression, alcoholism and elderly age. Here we report on the application of emerging nanopore sequencing methods to rapidly diagnose an atypical case of *C. canimorsus* septic shock.

**Case presentation:**

A 62 year-old female patient was admitted to an intensive care unit with septic shock and multi-organ failure six days after a reported dog bite. Blood cultures were unable to detect a pathogen after 3 days despite observed intracellular bacilli on blood smears. Real-time nanopore sequencing was subsequently employed on whole blood to detect *Capnocytophaga canimorsus* in 19 h. The patient was not immunocompromised and did not have any other known risk factors. Whole-genome sequencing of clinical sample and of the offending dog’s oral swabs showed near-identical *C. canimorsus* genomes. The patient responded to antibiotic treatment and was discharged from hospital 31 days after admission.

**Conclusions:**

Use of real-time nanopore sequencing reduced the time-to-diagnosis of *Capnocytophaga canimorsus* in this case from 6.25 days to 19 h. *Capnocytophaga canimorsus* should be considered in cases of suspected sepsis involving cat or dog contact, irrespective of the patient’s known risk factors.

**Electronic supplementary material:**

The online version of this article (10.1186/s12879-019-4173-2) contains supplementary material, which is available to authorized users.

## Background

Bloodstream infection can rapidly progress to septic shock; every hour without appropriate antibiotic therapy increases the risk of mortality while also increasing the length-of-stay in ICU. [Weiss 2014]. Use of broad-spectrum empirical antibiotics whilst awaiting the results of microbiological investigations increases selection pressure for multidrug-resistant organisms. Standard diagnostic methods rely on blood cultures followed by sub-culture, mass spectrometry or other confirmation methods to identify pathogens. Even with typical pathogenic bacteria, the turnaround ranges from 24 to 47 h with the use of mass spectrometry [1]. For fastidious organisms and those affected by antibiotic therapy, detection can be further delayed by several days or even result in false negative results [2, 3]. Furthermore viruses and some bacteria cannot grow in blood cultures.

To improve clinical outcomes in individuals with suspected bloodstream infection, new rapid diagnostics are essential. One promising approach is to leverage MinION sequencing (Oxford Nanopore Technologies, Oxford, UK) which sequences individual DNA or RNA molecules in an unbiased, real-time approach. The real-time sequence data capability can be coupled with downstream software to characterize the sequences as they are streamed off the instrument and has been demonstrated in a proof-of-concept study through the detection of Chikungunya, Ebola and Hepatitis C viruses in human blood [4], and lower respiratory tract infections [5].

**Fig. 1:**
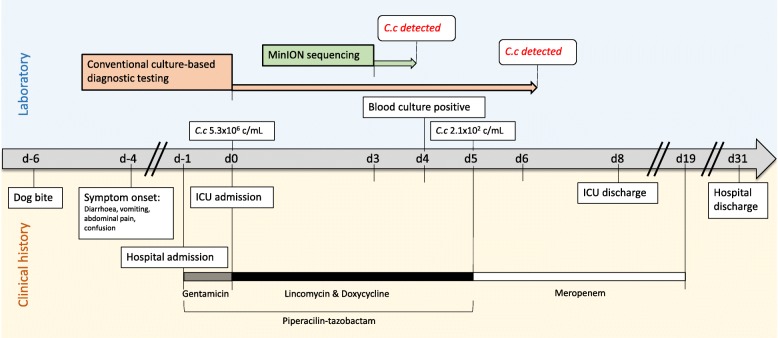
Timeline of patient’s clinical management progression, including timing of diagnostic testing, bacterial loads (genome copies/mL of blood) of *Capnocytophaga canimorsus* (*C.c*) in whole blood as determined by bespoke droplet digital PCR, and antimicrobial therapy. ICU admission is used as the day 0 reference point

Here we report an unusual case of *Capnocytophaga canimorsus* bloodstream infection where the MinION sequencer, along with droplet digital PCR (ddPCR) were used to rapidly detect and characterize the etiological pathogen (Fig. 1).

## Case presentation

A 62-year-old immunocompetent woman with no significant medical history presented to a regional emergency department with a three-day history of vomiting, diarrhea, non-specific abdominal pain and confusion. She reported a bite to her hand from her own dog five days prior. She denied alcohol use. She used topical diprosone 0.05% cream for dermatitis and oral esomeprazole 40 mg daily for indigestion. On initial examination she was afebrile, with a blood pressure of 90/60 mmHg, heart rate 110 beats/minute, and a Glasgow Coma Score of 15/15. She had right upper quadrant tenderness on abdominal examination and a petechial rash on her face and trunk.

A computerized tomogram of the abdomen demonstrated small volume ascites, marked gallbladder wall oedema, bilateral renal cortical necrosis and bilateral adrenal gland oedema. The spleen was present.

On presentation, her C-reactive protein was 359 mg/L (0–6); prothrombin time 39 s (8–14), activated partial thromboplastin time 122 s (22–35); serum creatinine 234 μmol/L (50–120); Alanine Transferase 869 IU/mL (0–45); Aspartate Aminotransferase (AST) 1199 IU/mL (0–41). Arterial blood gas examination demonstrated a pH 7.29 (7.35–7.45); pCO2 27.7 mmHg (32–48); pO2 75.2 mmHg (83–108); FiO2 estimated 0.28 based on oxygen therapy 2 L/min via nasal prongs; calculated Lactate 4.2 mmol/L (05.-2.2) and calculated base excess − 13.4 mmol/L.

A full blood examination demonstrated a haemoglobin of 156 g/L (115–160); platelets 13 × 10^9^/L (150–450); white blood cells 12.3 × 10^9^/L (4.0–11.0) and neutrophils 8.7 × 10^9^/L (2.0–7.5). There was prominent cytoplasmic vacuolation of the neutrophils, with some of the cells showing intracellular long rod-shaped inclusions (Additional file 2: Figure S1). The patient’s peripheral blood film did not demonstrate any Howell-Jolly bodies. HIV serology was negative. Extensive investigations including bone marrow aspirate and trephine biopsy did not demonstrate any cause of immunocompromise.

She was initially treated with 3 L intravenous fluid infusion; intravenous piperacillin-tazobactam 4.5 g and gentamicin 300 mg; intravenous hydrocortisone 100 mg and cryoprecipitate and fresh frozen plasma infusion. However she developed septic shock requiring intubation, mechanical ventilation and inotrope support. She was transferred to a large tertiary hospital for intensive care management.

Blood cultures collected upon admission to the intensive care unit remained negative after three days. The case was referred for enhanced testing using the MinION sequencer in response to a suspected bacterial aetiology due to the observation of rod-like neutrophil inclusions. Total nucleic acid was extracted from excess EDTA whole blood collected for routine diagnostic purposes on the day of admission (d0), followed by a 10-min rapid 1D library preparation (Additional file 1: Materials). The prepared sample was run on the MinION sequencer for 18 h, producing 17,552 good quality reads (206.2 Mbases, average read length 11,745 bp). Of note, yield was considered atypically low due to suboptimal pairing of library preparation kit and flow cell versions. The majority of reads (98.6%) were of human origin, with top microbial hits (Additional file 3: Figure S3) being for *Toxoplasma gondii* (43 reads, 604,353 bp total) and *Capnocytophaga canimorsus* (17 reads, 30,063 bp total). Further analyses of the *T. gondii* sequences indicated the detections were probable false positives attributable to likely contamination of the *T. gondii* reference genomes with human DNA (Additional file 1: Materials); a conclusion which was supported by further *T. gondii* PCR and serological testing (Additional file 1: Materials).

On day 4, the blood culture became positive, with subsequent sub-culturing showing small, slow-growing colonies with a distinct halo phenotype. On day 6, the sub-culture was identified as *C. canimorsus* by MALDI-TOF (bioMérieux, Australia). The isolate was β-lactamase-producing. The amoxycillin-clavulanate MIC by e-test (bioMérieux) was 0.032 mg/L and the ceftriaxone MIC 0.064 mg/L. Oral Stewart’s Media swabs from the offending dog were collected, but no *C. canimorsus* could be isolated due to rapid overgrowth by other commensal species.

Based on the generated *C. canimorsus* sequence data, a hydrolysis probe PCR assay was designed and used as a ddPCR to quantify the *C. canimorsus* bacterial load within the d0 and d5 whole-blood EDTA samples, as well as their associated plasma fractions (Additional file 1: Materials). The d0 whole blood sample showed approximately 150-fold greater concentration of bacterial load compared to the respective plasma (5.33 × 10^6^ copies/mL and 3.54 × 10^4^ copies/mL, respectively), while the d5 whole-blood and plasma showed equivalent, yet markedly reduced bacterial loads (2.1 × 10^2^ and 2.4 × 10^2^, respectively) (Additional file 4: Figure S4). The assay was also used in a real-time PCR format to detect *C. canimorsus* within two oral swabs collected from the dog (Additional file 1: Materials, Additional file 5: Figure S5).

The patient and dog *C. canimorsus* genomes were sequenced through a combination of MinION and Illumina sequencing, generating complete genomes which were nearly indistinguishable (Additional file 1: Materials). A computational analysis of virulence markers [6] indicated the patient isolate was of the Serotype D lineage. No antibiotic resistance markers were identified within the patient isolate genome, which fit the fully susceptible antibiotic profile obtained as part of the culture-based diagnostic investigation (Additional file 1: Materials).

The patient was treated initially with intravenous piperacillin-tazobactam, lincomycin and doxycycline, and subsequently with 14 days of intravenous meropenem (Fig. 1**)** which was chosen over a narrower spectrum non-Carbapenem beta-lactamase out of an abundance of caution due to the severity of the case and the experimental nature of the diagnostic test results. A percutaneous cholecystotomy was performed. Ionotropic and mechanical ventilation support was no longer required on day 3 of the ICU admission, concurrent to resolution of coagulopathy with the use of fresh frozen plasma. Liver function returned fully to normal by day 7, followed by resolution of thrombotic thrombocytopenic purpura (TTP) with platelet support on day 9. She required supportive care and intermittent haemodialysis which ceased approximately 7 weeks after presentation, but eventually returned home and remains well with essentially normal renal function.

## Discussion and conclusions

*Capnocytophaga canimorsus* is a common commensal bacteria of the oral cavities of cats and dogs which can lead to potentially fatal infections through minor bites, scratches or exposure to saliva [7, 8]. The fastidious bacteria requires extended incubation times, leading to delays in detection and appropriate treatment [7, 9]; thus new diagnostic approaches, such as the rapid nanopore sequencing described in this study, need to be considered.

In our case, the diagnosis of *C. canimorsus* bloodstream infection was considered based on the history of dog bite and the microsocopic appearance of rod-like inclusions in the neutrophils. Standard microbiological culture methods were initially negative, leading to the use of MinION sequencing which identified the bacteria in 19 h, in contrast to the 6.25 days it took for traditional culture-based methods to yield the same diagnosis. Rapid confirmation of the diagnosis allowed reduction of antibiotic use. There was initial concern that the presentation may represent haemolytic-uraemic syndrome consequent to a foodborne pathogen, and de-escalation of a public health response was possible with determination of the microbiological cause of the presentation.

The use of rapid sequencing and quantification technologies also allowed for additional characterization of the bacterial isolate, confirming the suspected etiological link between the dog bite and clinical presentation. Genomic analyses showed the isolate to be serotype D, which is not commonly found in clinical cases and thus not considered to be highly virulent [6]. The low virulent serovar status is unusual given the patient also did not have significant risk factors for *C. canimorsus* infection, such as immunosuppression, asplenia, heavy alcohol use, or elderly age [8, 10–12]. Unlike the atypical pathogen and patient phenotypes, the clinical presentation was mostly in line with common *C. canimorsus* sepsis features, including acute kidney injury [8, 9, 11, 13, 14], coagulopathy [13], TTP [8, 13], liver dysfunction [13, 14], and septic shock [8, 11, 13], as well as petechial rash [8, 14] and abdominal pain [8, 13, 14] upon presentation to hospital. The initial afebrile presentation however has been less frequently reported [7, 11, 13], while cholecystitis appears to be a rare, but not unheard of *C. canimorsus* complication [14, 15].

Apart from β-lactamase production, no additional resistance was detected by culture or molecular methods, consistent with the bacterial load reduction demonstrated by ddPCR, and the patient’s clinical response to antibiotic treatment. The initial antibiotic treatment regimen of pipercillin-tazobactam is expected to have been adequate for this particular infection given the bacterial isolate’s low MIC to amoxicillin-clavulanate. The observed bacterial load reductions highlight the importance of obtaining blood samples prior to, or early in the application of antibiotics for sequencing-based diagnostic approaches, as the large decrease in bacterial DNA seen on d5 would reduce the effective sensitivity or even lead to outright false negative results with the MinION platform. While not directly evaluated, the bacterial load (5.3 × 10^6^ copies/mL) and resulting number of *C. canimorsus* nanopore reads (17) show that sample preparation, such as reduction of human DNA or bacterial concentration, will be necessary to obtain sensitivities approaching that of a real-time PCR (ie: approximately 1000–500 copies/mL). Additionally, the ddPCR data show the utility of using whole-blood rather than plasma for PCR or sequencing based approaches to sepsis diagnosis given the observed high intracellular bacterial loads. Currently the MinION output formats are still mostly research-centric, however efforts are underway to make the reporting more streamlined and less ambiguous in order to make the platform amenable for use in the routine diagnostic setting. Likewise, the logistics for running reflexive diagnostic testing with the MinION is still maturing, yet sample and library preparation as well as the sequencing can already be used with standard routine molecular diagnostic laboratory equipment. The cost (approximately $1000 AUD at the time of writing) still limits the diagnostic applicability to specialised enhanced testing, however the recent release of the MinION Flongle disposable flow cell promises to decrease the testing costs by about 5-fold.

In summary, *Capnocytophaga canimorsus* should be considered in cases of suspected sepsis involving cat or dog contact, even if the patient does not present with known risk factors. Real-time nanopore sequencing has a demonstrated ability to greatly reduce the time to diagnosis, particularly in cases of atypical infections caused by fastidious organisms, and shows potential for accurate pathogen characterization to inform clinical and public health management.

## Additional files

Additional file 1: Materials. File documenting in detail the methods and results of the enhanced molecular testing and investigations. **Table 1.** PCR primer and probe and corresponding synthetic control sequences designed against the *C. canimorsus* sequences generated from the initial whole-blood nanopore sequencing run. Highlighting indicates oligo targeting in the synthetic control. **Figure S2**. MinION sequencing time and *C. canimorsus* read count plot from initial d0 EDTA blood sample. (DOCX 78 kb)

Additional file 2: **Figure S1**. Gram stain of blood smear showing intracellular bacilli (red arrows). (JPG 77 kb)

Additional file 3: **Figure S3**. One Codex output from submission of the whole-blood nanopore good quality read sequences showing characterized microbial reads within the sample. (JPG 54 kb)

Additional file 4: **Figure S4**. Droplet Digital PCR results of the bespoke *C. canimorsus* assay showing A) positive and negative droplet counts and B) calculated absolute quantification of the target *C. canimorsus* template. (PDF 144 kb)

Additional file 5: **Figure S5**. Results of the bespoke *C. canimorsus* real-time PCR assay with duplicate reactions of the two dog oral swabs (green and orange) and synthetic positive control (red). Purple indicates negative control. (JPG 152 kb)

## Abbreviations

ddPCR: droplet digital PCR
EDTA: Ethylenediaminetetraacetic acid
HIV: Human Immunodeficiency Virus
ICU: Intensive Care Unit
MALDI-TOF: Matrix Assisted Laser Desorption/Ionization Time of Flight
