# Supplementary material for: Rapid diagnosis of Capnocytophaga canimorsus septic shock in an immunocompetent individual using real-time Nanopore sequencing: a case report
Source: BMC Infect Dis. 2019 Jul 24;19:660. doi: 10.1186/s12879-019-4173-2 (PMC6657077; doi:10.1186/s12879-019-4173-2)
Supplement: Supplementary file 1 — Materials. File documenting in detail the methods and results of the enhanced molecular testing and investigations. Table 1. PCR primer and probe and corresponding synthetic control sequences designed against the C. canimorsus sequences generated from the initial whole-blood nanopore sequencing run. Highlighting indicates oligo targeting in the synthetic control. Figure S2. MinION sequencing time and C. canimorsus read count plot from initial d0 EDTA blood sample. (DOCX 78 kb) [file 12879_2019_4173_MOESM1_ESM.docx]

**Supplementary Materials – “Rapid Diagnosis of *Capnocytophaga canimorsus* Septic Shock in an Immunocompetent Individual Using Real-Time Nanopore Sequencing: A Case Report”**

**Patient Involvement**

Ethics approval for this case report was waived by the Metro North Hospital and Health Service Human Research Ethics Committee, in line with the Committee’s guidelines for single Case Reports. The patient’s written consent was obtained to conduct and publish this case study.

**DNA Extraction**

Excess whole-blood EDTA samples collected for routine diagnostic purposes from the day of admission (d0) and 5 days post—admission (d5) were split, with one aliquot of each blood being centrifuged to obtain the serum fraction. A 10ml aliquot of the blood culture which flagged positive on day 4 was also obtained and processed either by centrifugation or pre-heating at 98°C for 5 minutes followed by centrifugation, with the supernatants being subsequently used for nucleic acid extraction. 200µl of each sample was extracted using the QIAamp DNA Blood Mini kit (QIAGEN, Australia) as per manufacturer’s instructions. DNA yield and quality measured with the Qubit HS DNA kit (Thermo Fisher, Australia) and the Nanodrop (Thermo Fisher, Australia) indicated that the yield and DNA purity was greatest in the d0 followed by the d5 whole blood (20.4 ng/µl; 1.8 (260/280) and 19.7ng/µl; 1.84 (260/280), respectively), while both the centrifuged and heated blood culture supernatants produced low DNA yields or extracts of low purity (5.2ng/µl; 0.24 (260/280) and 0.47ng/µl; 1.81 (260/280), respectively).

**Whole-blood nanopore sequencing**

The rapid sequencing kit (SQK-RAD003 version RSE_9040_v1_revC_04Jul2017, Oxford Nanopore Technologies, UK) was used for library preparation of the MinION sequencing run. In brief, a transposase with adaptors was added to the sample (2 minute reaction) and the nanopore adaptors were ligated to the tagmented DNA (5 minute reaction). A few modifications of the protocol were needed to achieve a better sequencing performance: 4uL of fragmentation enzyme (FRA) were added to 11µL of 800ng of template DNA; 2µL of rapid adaptor (RPD) were used instead of 1µL and 17µL of library (800ng) was loaded into the FLO-MIN107 flow cell (version R9.5.1). The run was performed in a MinION MK1B for 18hours with the protocol script “SQK-RAD003” chosen from the MinKNOW software (v1.10.23).

The MinION run outputted 36,000 reads with 226,184,903 events in total and the raw data was base called with Albacore (v2.1.3) using the following workflow: FLO-MIN107, SQK-RAD003 and configuration file: r95_450bps_linear.cfg. Out of the total fastq files produced, 17,552 reads were classified as pass in this software. All pass reads were aligned to the in-house genomes database by minimap2 [1] and then analysed by the real-time species typing module from Japsa package v1.7-10a [2]. In total, 17 reads (additional supplementary file “Capnocytophaga canimorsus MinION reads.fa”) were classified as *C. canimorsus* in origin, with the majority of reads being detected in the first 100 minutes (Supplementary Figure 2).

Analyses results were further confirmed by uploading the “pass” reads onto the One Codex online portal [3] (One Codex, USA). Briefly, the One Codex algorithm removes reads matching human sequences prior to characterising the non-host reads using a proprietary whole-genome database containing over 80,000 bacterial, viral, fungal and protist species and strains. The One Codex results (**Supplementary Figure 3**) showed a similar number of *Capnocytophaga canimorsus* reads being characterised, however also showed a number of other matches, most prominent of which was to *Toxoplasma gondii*. The majority of minor read hits were considered reagent or laboratory contaminants as they were originating from environmental organisms. The *T. gondii* reads all aligned to the unplaced scaffolds of the *T. gondii* ME49 reference genome (RefSeq Accession GCF_000006565.2), while smaller fragments (eg: 1000-2000bp) of the reads would consistently align with existing human sequences. Furthermore, routine diagnostic results showed that the patient was IgG, but not IgM seropositive, as well as *T. gondii* not being detected by real-time PCR in their d0 and d5 whole blood. Thus, due to the multiple lines of evidence, we considered the *T. gondii* hits to be spurious and most likely to be of human origin. This would also suggest that human sequences have contaminated the *T. gondii* reference genome during its production and sequence assembly, a proposition supported by previous findings [4].

**Culture isolate antibiotic susceptibility**

The isolate was β-lactamase-producing. The amoxycillin-clavulanate MIC by e-test (bioMérieux) was 0.032 mg/L and the ceftriaxone MIC 0.064 mg/L.

**Culture isolate nanopore sequencing**

The *C. canimorus* sub-culture was collected and resuspended in PBS, prior to being extracted using the QIAamp DNA Blood Mini kit and eluted into 50µl of water. The library preparation for the isolate was done following the 1D Native barcoding genomic DNA (EXP-NBD103 version: NBE_9006_v103_revP_21Dec2016 and SQK-LSK108, Oxford Nanopore Technologies, UK) protocol with the following modifications: 1.5µg input DNA, 10uL of end-repair reaction buffer and 4.5µL of the end-repair enzyme mix NEBNext Ultra II End-repair/dA-tail module (New England BioLabs, USA); 5 µL of native barcoding 1 (NB01) and 25µL of Blunt/TA Ligase Master mix (New England BioLabs, USA); 30µL of barcode adapter mix (Oxford Nanopore Technologies, UK), 30µL of NEBNext Quick Ligation Reaction Buffer (5x) (New England BioLabs, USA) and 15µL of Quick T4 DNa Ligase (New England BioLabs, USA). 15µL (501ng) were loaded into the flow cell (FLO-MIN107 version R9.5.1).

The run was performed in a MinION MK1B for 4.5hours with the protocol script “SQK-LSK108” chose from the MinKNOW software (v1.10.23).

The nanopore output was 1,323,508 reads with 1,356,630,003 events in total. Basecalling was accomplished using the following Albacore (v2.1.3) workflow: FLO-MIN107, SQK-LSK108, configuration file: r95_450bps_linear.cfg, and “—barcoding” option. Of the 948,048 reads classified as “pass” in this software, 772,628 reads were assigned to be from *C. canimorsus* using One Codex. Canu version 1.7 [5] was applied for genome assembly on the nanopore data, resulting in a draft genome that has 97.12% average identity to the reference *C.canimorsus* Cc5 (RefSeq GCF_000220625.1). The draft assembly was polished by nanopolish version 0.9.0, making the figure slightly improved to 97.59%.

**ddPCR and real-time PCR**

Based on the initial whole blood *C. canimorsus* reads, a dual-labeled hydrolysis probe PCR assay was designed with the help of the Geneious 9.1.8 software package (Biomatters Ltd, New Zealand) by targeting conserved sequences shared with four other *C. canimorsus* reference strains (CP022382, CP022389, CP002113, and CP022388) (Supplementary Table 1). Specificity of the assay was tested within the Geneious software as well as through NCBI BLASTn. A double stranded linear DNA synthetic control specific to the assay was also designed (Integrated DNA Technologies, USA) (**Supplementary Table 1**). The PCR assay was adapted for use in droplet digital PCR (ddPCR) on the QX-200 ddPCR system (Bio-Rad, Australia). Briefly, 12.5µl of ddPCR Supermix for Probes (Bio-Rad, Australia), 10pmol of each primer, 4pmol of probe and 6.25µl of DNA extract were combined into a final 25µl reaction volume, with droplet generation performed on the QX-200 companion automated AutoDG platform. PCR was performed in a deep-well C-1000 PCR cycler (Bio-Rad, Australia) under the following conditions: incubation at 95°C for 10 minutes, followed by 40 cycles of 95°C for 30s, 55°C for 60s and 72°C for 60s, with a final extension step at 98°C for 7 minutes. Reaction volume for the cycling parameters was set at 40µl. Both of the whole blood and the equivalent plasma DNA extracts were run alongside a 1:20 isolate sub-culture extract and two serial dilutions of the synthetic control (10^-7^ and 10^-8^) resuspended in purified human DNA as the positive controls, and purified human DNA as the negative control and baseline threshold determinant. All DNA extracts including the human DNA were centrifuged through Covaris G-Tubes (Covaris, USA) at 8,000g.

All three positive controls were successfully amplified, with the two serially diluted synthetic controls retaining the logarithmic scale on the resulting absolute quantification results (Wells G01 and H01, **Supplementary Figure 4**), while the 1:20 culture (Well E01) went beyond the upper limit of sensitivity and saturated the positive droplet count. The negative control (Well F01) did not show non-specific amplification, while all four blood samples produced positive amplification (Wells A01-D01, **Supplementary Figure 4**).

Two swabs were taken of the offending dog’s oral cavity before being placed in Stewart’s Transport Media. DNA isolation from the swabs involved processing the swab heads with the AllPrep PowerViral DNA/RNA extraction kit (QIAGEN, Australia) as per manufacturer’s instructions and included the bead-beating step of the snapped-off swab heads. The bespoke PCR assay was adapted to real-time PCR use and applied to both oral swab extracts as well as the 10^-7^ synthetic control dilution. Briefly, the real-time PCR consisted of 10µl SensiMix Probe II PCR mix (Bioline, Australia), 8pmol of primer, 3.2pmol of probe, 2µl of template made up to a total 20µl reaction volume with the following cycling conditions: 95°C incubation for 10 minutes, and 45 cycles of 95°C for 15s and 60°C for 60s. Despite low total DNA concentrations extracted from both swabs (0.9ng/µl and 1.16ng/µl), *C. canimorsus* could be detected consistently at a mean Ct value of 22.9 (**Supplementary Figure 5**).

**Illumina sequencing**

Additional sequencing was undertaken to complete the cultured isolate genome along with the dog swab extract. Both sample extracts were prepared into NexteraXT libraries and subjected to Illumina NextSeq500 2x150bp sequencing. The direct sequencing of the dog swab extract yielded 32 713 374 reads, of which 4 200 000 reads mapped to the *C. canimorsus* genome (NC_015846), giving an average sequence depth of 243 times, while the isolate produced 3 271 374 reads, which represents an average sequence depth of 215 times.

The genome for the patient *C. canimorsus* isolate was completed and generated one circular contig 2555606 bp in size. This comprised of 2359 genes, 9 rRNAs (3 5S, 3 16S, 3 23S), 44 tRNAs, 3 ncRNAs and 126 pseudo genes. Annotation was performed using the NCBI Prokaryotic Genome Annotation Pipeline.

**Genome analyses**

The patient isolate’s genome was screened computationally (Geneious 9.1.8 software package (Biomatters Ltd, New Zealand) for the presence of 6 PCR targets (SeroA, SeroB, SeroC, SeroD, SeroE and SeroABC) defined by Hess et al [6] which were found to be serovar markers, including the more virulent A, B and C serovar forms. Of the 6 PCR targets, only the SeroD primer targets were present on the patient genome, producing a hypothetical PCR product of 590bp, in line with the Hess et all results. The genome was also analyzed for acquired antimicrobial resistance genes using the ResFinder 3.0 online portal [7], but did not detect any markers even when using a low threshold (sequence identity: 30%, minimum length: 20%). The ResFinder results correlated with those obtained through routine culture-based diagnostic testing of the patient *C. canimorsus* using the e-test (bioMérieux, Australia) which did not identify any clear antibiotic resistance. To determine the SNP difference between dog and human clinical isolate, Illumina reads (dog) were mapped to the assembly genome (clinical isolate) using BWA-MEM [8]. Three SNPs between the clinical and dog isolates were detected at positions 113366 (C🡪A), 706280 (C🡪A), and 1269042 (A🡪T). The GATK [9] pipeline was utilized to call SNPs, as previously described [10].

**Data availability**

Whole genome sequencing for the patient (Cc_RBWH_01c, BioSample: SAMN10160287) and dog (Cc_RBWH_01d, BioSample: SAMN10169587) *C. canimorsus* isolates has been deposited under BioProject [PRJNA494285](https://www.ncbi.nlm.nih.gov/bioproject/PRJNA494285) (www.ncbi.nlm.nih.gov/bioproject/[PRJNA494285](https://www.ncbi.nlm.nih.gov/bioproject/PRJNA494285)). Illumina and Nanopore sequencing data has been deposited on the Sequence Read Archive ([www.ncbi.nlm.nih.gov/sra/](http://www.ncbi.nlm.nih.gov/sra/)). Accession numbers are as follows: Cc_RBWH_01c (Illumina: SRR7957427; Nanopore: SRR7957428, complete genome: CP032681) and Cc_RBWH_01d (Illumina: SRR7957431). Sequences to be released upon acceptance of manuscript.

**Supplementary References**

1. Li H. Minimap2: pairwise alignment for nucleotide sequences. *Bioinformatics* 2018; 10:1-7.
2. Cao MD, Ganesamoorthy, D, Elliott AG, et al. Streaming algorithms for identification of pathogens and antibiotic resistance potential from real-time MinION sequencing. *GigaScience* 2016; 5:32.
3. Minot, SS, Krumm N, Greenfield NB. One codex: a sensitive and accurate data platform for genomic microbial identification. *bioRxiv* 2015 [**Preprint**]; Sept. 25, 2015 [cited 24 Oct. 2018]. Available at: <https://doi.org/10.1101/027607>.
4. [Kryukov K](https://www.ncbi.nlm.nih.gov/pubmed/?term=Kryukov%20K%5BAuthor%5D&cauthor=true&cauthor_uid=27611326), [Imanishi T](https://www.ncbi.nlm.nih.gov/pubmed/?term=Imanishi%20T%5BAuthor%5D&cauthor=true&cauthor_uid=27611326). Human Contamination in Public Genome Assemblies. [*PLoS One*](https://www.ncbi.nlm.nih.gov/pubmed/27611326) 2016; 11:e0162424.
5. Koren S, Walenz BP, Berlin K, et al. Canu: scalable and accurate long-read assembly via adaptive k-mer weighting and repeat separation. *Genome Res* 2017; 27:722-736.
6. Hess E, Renzi F, Koudad D, et al. Identification of virulent Capnocytophaga canimorsus isolates by capsular typing. *J Clin Microbiol* 2017; 55:1902–1914.
7. Zankari E, Hasman H, Cosentino S, et al. Identification of acquired antimicrobial resistance genes. J Antimicrob Chemother 2012; 67:2640-4.
8. Li H. Aligning sequence reads, clone sequences and assembly contigs with BWA-MEM. arXiv 2013 [**Preprint];** May 26 2013 [cited 24 Oct. 2018]. Available at: https://arxiv.org/abs/1303.3997 .
9. McKenna A, Hanna M, Banks E, et al. The Genome Analysis Toolkit: a MapReduce framework for analyzing next-generation DNA sequencing data. Genome Res 2010; 20:1297–1303.
10. Bainomugisa A, Lavu E, Hiashiri S et al. Multi-clonal evolution of multi-drug-resistant/extensively drug resistant Mycobacterium tuberculosis in a high-prevalence setting of Papua New Guinea for over three decades. Microb Genom 2018, [**Ahead of Print]**, doi: 10.1099/mgen.0.000147.

**Supplementary Tables and Figures**


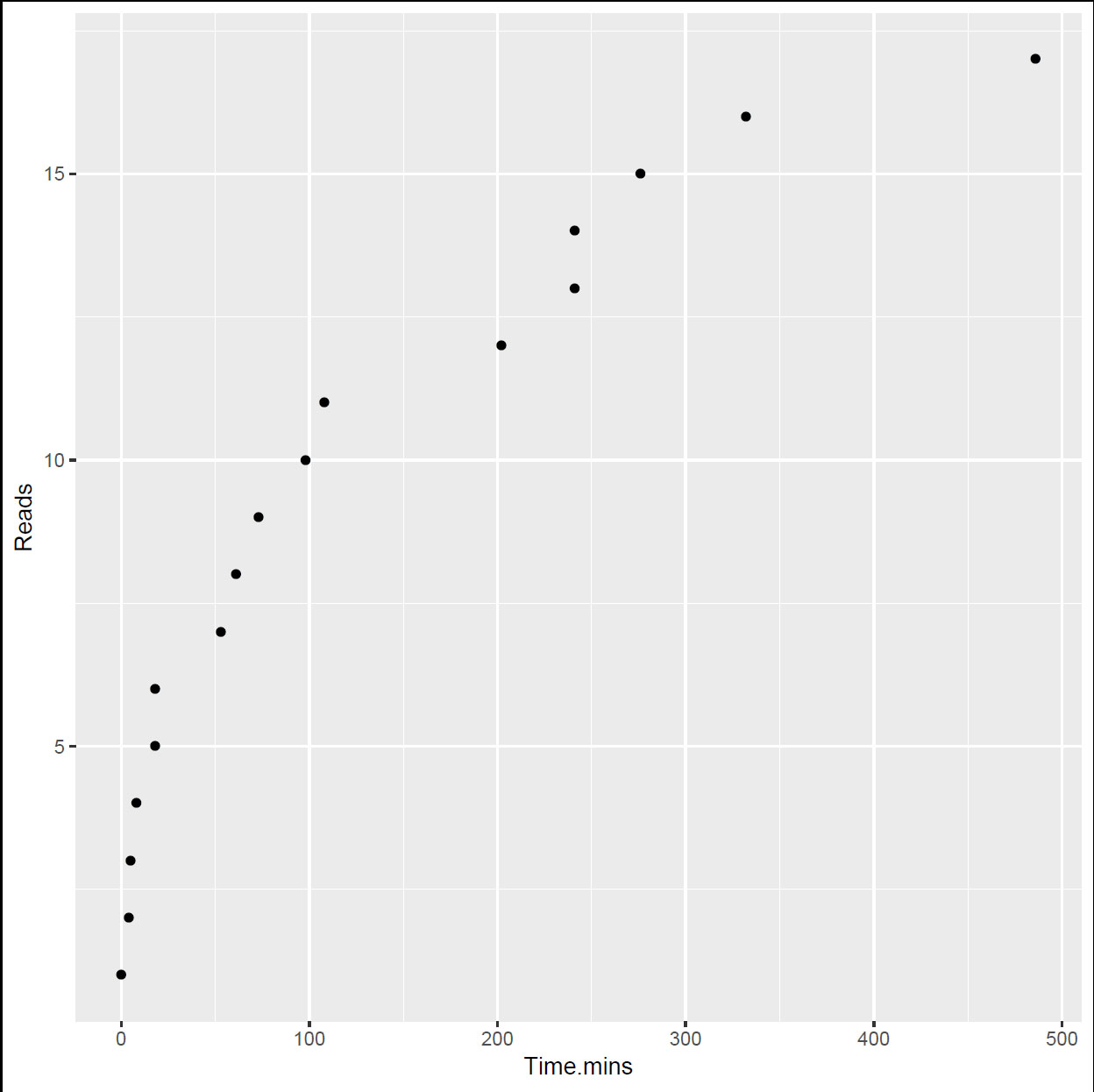


**Supplementary Figure 2.** MinION sequencing time and *C. canimorsus* read count plot from initial d0 EDTA blood sample.

**Supplementary Table 1.**  Real-time/ddPCR oligo (5’-3’) sequences and associated synthetic control.

**Name Sequence (5’-3’)**

C.cani_MinIon_F: GGGCTACGTACTGATTGAGC

C.cani_MinIon_R: TTCCTTCTAACAAACATTCGGTAT

C.cani_MinIon_FAM: FAM-AGGTTGCCACAGACCCAGAAAACA-BHQ1

C.cani_MinIon_synth:

GGGATTACTACAACCGTTGCCGAAAAAATAGCCACATTGTAAATAGCAATATCAGGGAGGGCTACGTACTGATTGAGCCTAAACTTATCAATATCCACCAACAAGGTTGCCACAGACCCAGAAAACACTACAAAGCCATATCACCACAATACCGAATGTTTGTTAGAAGGAAATCTATATGAAACTGAGGGTCGAACATAAAAATGCAAAACAAACATA
