# Supplementary figures and images for: Rapid diagnosis of Capnocytophaga canimorsus septic shock in an immunocompetent individual using real-time Nanopore sequencing: a case report
Source: BMC Infect Dis. 2019 Jul 24;19:660. doi: 10.1186/s12879-019-4173-2 (PMC6657077; doi:10.1186/s12879-019-4173-2)

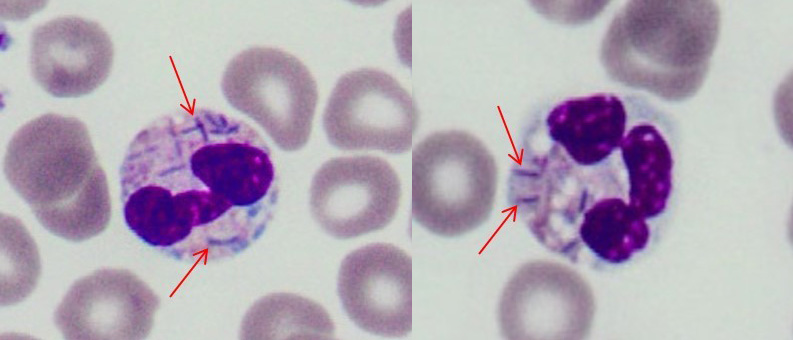

Supplement: Supplementary file 2 — Figure S1. Gram stain of blood smear showing intracellular bacilli (red arrows). (JPG 77 kb) [file 12879_2019_4173_MOESM2_ESM.jpg]

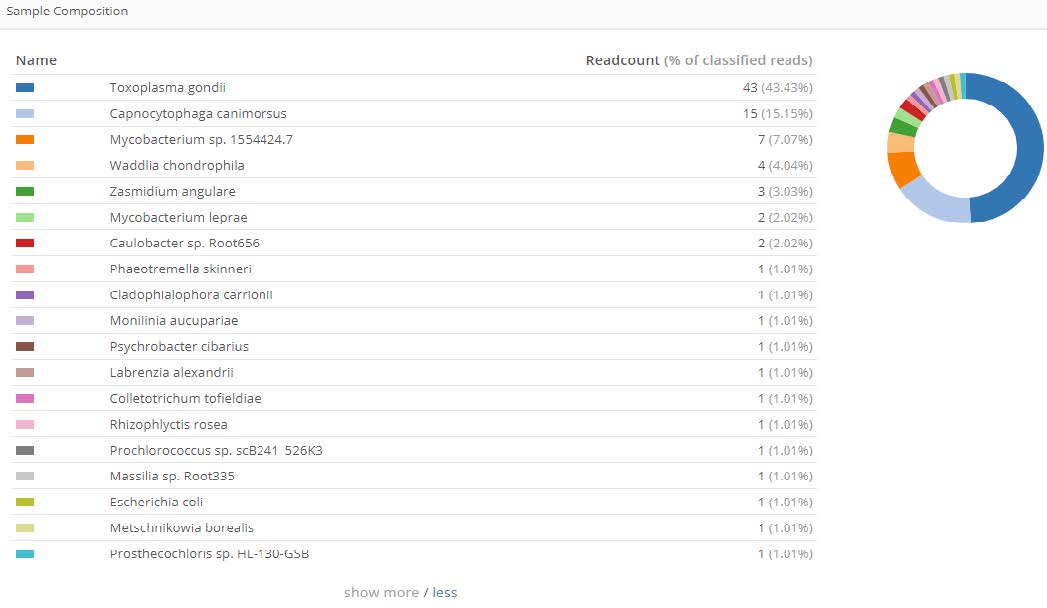

Supplement: Supplementary file 3 — Figure S3. One Codex output from submission of the whole-blood nanopore good quality read sequences showing characterized microbial reads within the sample. (JPG 54 kb) [file 12879_2019_4173_MOESM3_ESM.jpg]

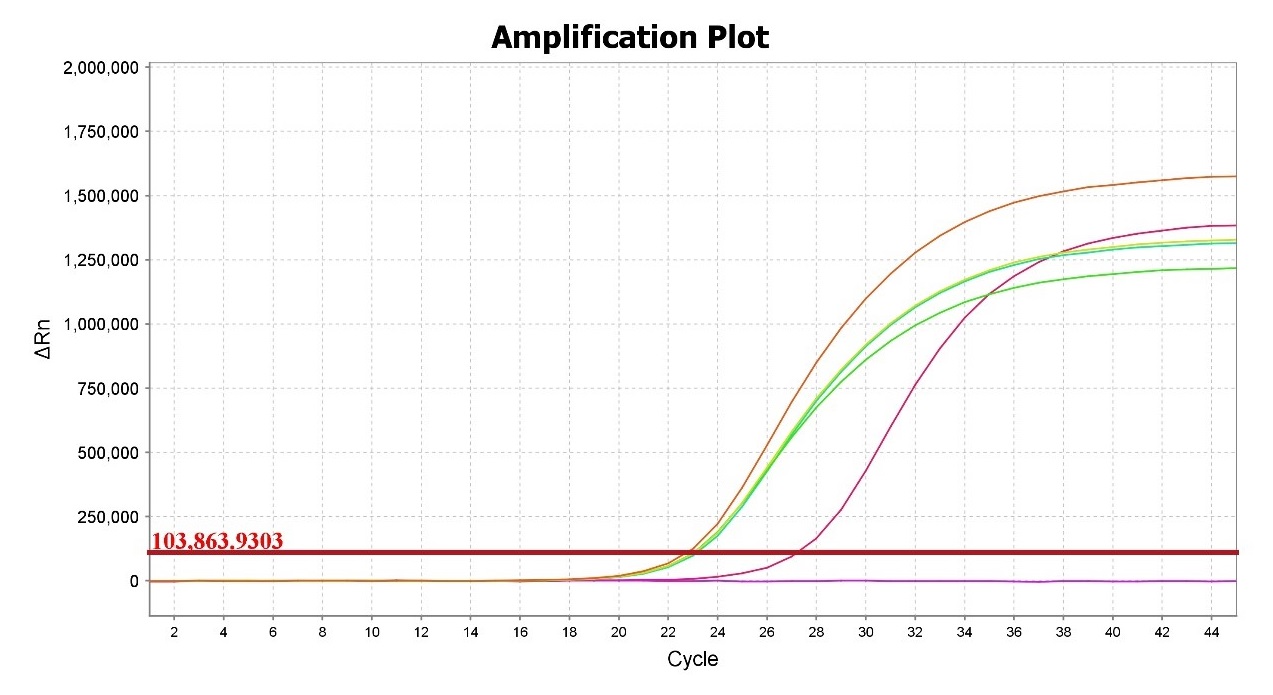

Supplement: Supplementary file 5 — Figure S5. Results of the bespoke C. canimorsus real-time PCR assay with duplicate reactions of the two dog oral swabs (green and orange) and synthetic positive control (red). Purple indicates negative control. (JPG 152 kb) [file 12879_2019_4173_MOESM5_ESM.jpg]
