# Supplementary material for: Rapid diagnosis of Capnocytophaga canimorsus septic shock in an immunocompetent individual using real-time Nanopore sequencing: a case report
Source: BMC Infect Dis. 2019 Jul 24;19:660. doi: 10.1186/s12879-019-4173-2 (PMC6657077; doi:10.1186/s12879-019-4173-2)
Supplement: Supplementary file 4 — Figure S4. Droplet Digital PCR results of the bespoke C. canimorsus assay showing A) positive and negative droplet counts and B) calculated absolute quantification of the target C. canimorsus template. (PDF 144 kb) [file 12879_2019_4173_MOESM4_ESM.pdf]

A)

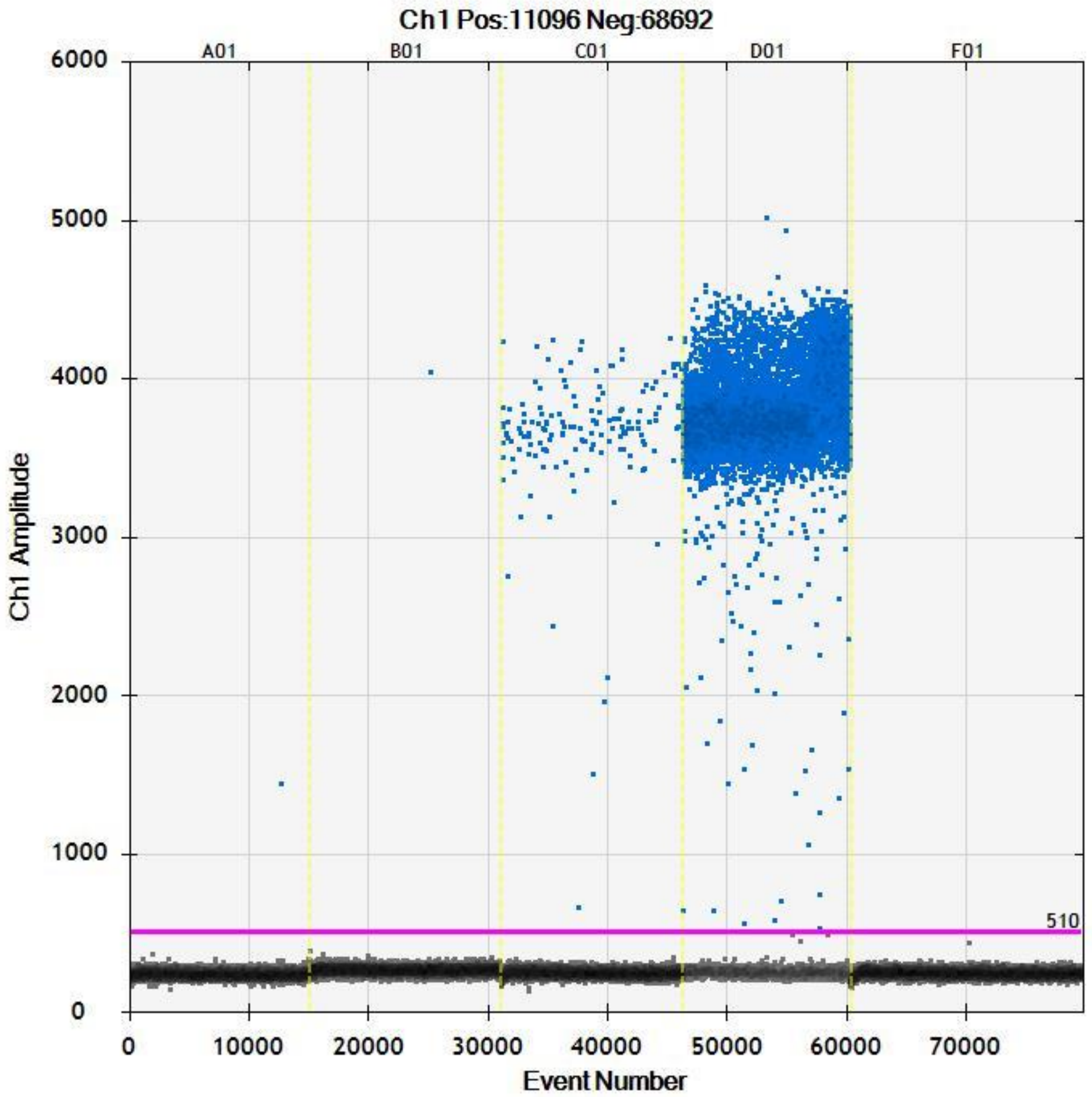

B)

Well	Sample	copies/µl extract (5µl/reaction)	copies/mL of sample
A01	d5 Plasma	0.32	240
B01	d5 EDTA	0.28	210
C01	d0 Plasma	47.2	35400
D01	d0 EDTA	7116	5337000
E01	*C. canimorsus* 1:20 culture	Saturated	n/a
F01	Human DNA	0	n/a
G01	*C. canimorsus* synth -7	2308	1731000
H01	*C. canimorsus* synth -8	192.8	144600
